# The Role of Religion and Religiosity in Health-Promoting Care for the Body During the Lockdowns caused by the COVID-19 Pandemic in Egypt, Poland and Romania

**DOI:** 10.1007/s10943-022-01624-3

**Published:** 2022-08-13

**Authors:** Małgorzata Lipowska, Arkadiusz Modrzejewski, Artur Sawicki, Mai Helmy, Violeta Enea, Taofeng Liu, Bernadetta Izydorczyk, Bartosz M. Radtke, Urszula Sajewicz-Radtke, Dominika Wilczyńska, Mariusz Lipowski

**Affiliations:** 1 Institute of Psychology, University of Gdańsk, Bażyńskiego 4, 80-309 Gdańsk, Poland; 2 Institute of Political Sciences, University of Gdańsk, Gdańsk, Poland; 3 Psychology Department, College of Education, Sultan Qaboos University, Muscat, Oman; 4 Psychology Department, Faculty of Arts, Menoufia University, Shibīn al-Kawm, Egypt; 5 Department of Psychology, Faculty of Psychology and Education Sciences, “Alexandru Ioan Cuza” University, Iasi, Romania; 6 School of Physical Education, Zhengzhou University, Zhengzhou, China; 7 Institute of Psychology, Jagiellonian University, Krakow, Poland; 8 Laboratory of Psychological and Educational Tests, Gdańsk, Poland; 9 Specialist Psychological and Educational Consulting Centre, Gdańsk, Poland; 10 Department of Psychology, Gdańsk University of Physical Education and Sport, Gdańsk, Poland

**Keywords:** SARS-CoV-2, Eating habits, Physical activity, Sunni Islam, Roman Catholicism, Orthodox Christianity

## Abstract

The coronavirus pandemic (COVID-19), as a widespread health threat, has triggered an increase in health-related behaviours, both pro-and anti-health, especially with regard to diet and physical activity. One of the factors modifying the intensity of such activities may be the religious doctrine and religiosity with which a person is associated. A total of 1502 people (1147 women) from countries that feature one dominant religion, took part in the study. Participants represented Sunni Islam (Egypt, *n* = 798), Roman Catholicism (Poland, *n* = 443) and Orthodox Christianity (Romania, *n* = 261). The Coronavirus Anxiety Scale, the Eating Attitudes Test and the Inventory of Physical Activity Objectives were used in the study. Fear of COVID-19 is associated with engagement in pro-health activity, although not to such a significant extent as might be expected. The type of religion in question was revealed to moderate this relationship, but the intensity of religiosity was not found to serve as a moderator.

## Introduction

### Religious Perceptions of the Body and Corporeality

Religion and morality are closely related phenomena in social life. Even if religion is not a condition of morality in the philosophical sense, it affects the moral attitudes of believers, determines their behaviour and affects assessments of these behaviours in particular (Norenzayan, 2014). Especially in traditional societies, a strong correlation between morality and religious faith can be observed, at least on a descriptive level (McKay & Whitehouse, 2015). The view of corporality by various religious systems is an important and complex issue. The body is subject to moral norms, prescriptions and proscriptions, which are often detailed and which do not merely relate to the sexual sphere but also to so-called ritual purity, as in the cases of Judaism and Islam. The body and corporeality typically play a key role in the religious concept of morality (e.g., Catholic moral theology). Religious morality must not always be grounded in theological reflection; more often, such morality is simply practised. To some extent, this morality can even be syncretic when religious beliefs are combined with local cultures and customs. In this context, the problem of the complexity of the issue of corporeality in religious morality emerges. Apart from small religious communities that can be characterised by a relatively unitary moral doctrine, world religions are distinguished by certain degree of pluralism, which is not only theological but also moral. Even if some moral practices incur the highest degree of sanctions, as in the case of the so-called revelatory scriptures of monotheistic religions, the ways in which these practices are interpreted can differ and vary not only in accordance with the particular branch of religion that one professes but also within one single denomination or confession, in which the same practice can be the subject of various theological or ethical interpretations (Weaver, 2020). Although certain denominations, such as the Catholic Church, have hierarchical structures that include a centre that is officially responsible for religious doctrine and plays a monopolistic role in the fields of theology and morality (in the case of Catholicism, i.e. the pope), in terms of theological and moral debate, there remains a discourse in which moral issues, including issues relating to corporeality, are understood and interpreted in various ways (Curran, 2019). Therefore, it is not practical to seek a general pattern of morality relating to the body and corporeality that could be characterised as a distinctive element of the moral theology of a given religion or of morality as practised by the followers of a religion (or even simply in the context of one denomination within a religion). At most, it is possible to highlight general doctrinal assumptions or the most stereotypical understanding of the body and corporeality among the followers of a given religion, an understanding which is naturally not static but which evolves and takes on new forms constantly. This understanding also depends on the particular cultural context in which a religion is practised.

### Health-Promoting Care for the Body in Christianity and Islam

Any behaviour that has an effect on the health of an individual or society is called a health-related behaviour or a health behaviour. Health-related activities are described as “…reactive, habitual and/or purposeful activities of a person, which are based on the objective knowledge of health and subjective conviction—in an important, mutual relation with health” (Heszen-Celińska & Sęk, 2020). Health activities include activities that promote and help to maintain health as well as activities that are impede health and are harmful. The most typical health-promoting behaviours are regular physical activity (PA) and a balanced diet (WHO, 2020). Both physical activity and a healthy diet have a direct impact on the condition and health of the body as well as indirect impacts on mental health and general well-being (Owen & Corfe, 2017). Taking care of one’s health is one of the most important components of a healthy lifestyle. Such a lifestyle includes various behaviours as well as attitudes and a general philosophy towards an individual’s life; thus, this activity depends on the social and cultural environment in which a person lives as well as on their beliefs and the hierarchy of values and religious doctrines with which that person is associated (Abdala et al., 2021). Because religious doctrines take different approaches to the body and corporeality, the value attributed to health-promoting behaviours such as physical exercise and diet may vary. Let us consider the issues of the body, corporeality, and sport with respect to the two global religions that are central to our study, namely, Christianity and Islam.

#### Christianity

Christianity (in this context, only European Christianity, which developed on Latin and Greek-Byzantine soil) has expressed a great deal of ambivalence towards the body and corporeality since its very beginning (Jacobson et al., 2016). On the one hand, this religion intended to overcome Jewish orthopraxy related to the observance of rules related to the maintenance of ritual purity and the appreciation of the body via the incarnation and resurrection of the Son of God as well as via the Christian belief in the resurrection of the body, which was not common among Jews, as well as the very notion of the embodiment of God, which was almost impossible to comprehend. On the other hand, the body, especially in the context of sexuality, was viewed primarily as a source of sin as a result of the moral teachings of the Apostle Paul and the Manichean themes that were present in the doctrines of ancient Christian thinkers. Asceticism and sexual restraint became the model for traditional Christian morality, while virginity and celibacy became the ideal of a godly life (van den Heever, 2014). Although Christianity, in its incorporation of Greek philosophy, did not adopt the Greek ideal of corporeality, the standards of beauty that were characteristic of ancient Greece and Rome have been present in Christian art since its beginnings. The figure of Christ as a good shepherd and especially that of his naked suffering body on the cross indicate the anatomical beauty of an athletic man. The realism or even naturalism of Christian art demonstrates the value of the body and determines its apotheosis. Christ’s body, as depicted in sculptures and paintings, has all the features of the human body (Williamson, 2004). Therefore, the human body, although it is occasionally treated as a source of sin, is also the subject of concern. Asceticism is not an end in itself but rather a way of achieving spiritual union with God (Jonveaux, 2012). Christianity gradually rejected all forms of self-mutilation as manifestations of excessive asceticism; mutilation of the body has become the hallmark of heretical sects rather than of mainstream Christianity. Caring for the bodily aspect of human existence became a moral imperative, and any manifestations of self-harm in Christianity were rejected.

As with the body, the attitudes of Christian thinkers towards sport were ambivalent for many centuries. Excessive concentration on the flesh was thought to divert the attention of the faithful from the spiritual realm. Sports rivalries were associated with the bloody Roman Games rather than the Greek pattern of physical culture. However, as early as the villages of the Middle Ages, sports had already become an integral part of church life. Nevertheless, care for the physical form was still not viewed as a Christian activity, a perspective to which the Reformation and especially the advent of Puritanism also contributed (Shilling & Mellor, 2014). It was not until the twentieth century that sport became “Christian” from the perspectives of both Catholics and Protestants. This re-evaluation occurred as a result of the reinterpretation of the theological and philosophical thought of St. Thomas and the related overcoming of the Platonic anthropology that depreciates the body, which is understood as a prison of the soul. The popularity of sport among Christians was influenced by the attitudes of various popes, especially Pius XII and John Paul II, who were great fans of sport, and the development of Christian sports organisations in both Catholic and Protestant contexts (Watson & Parker, 2013). Attitudes towards sports have also evolved among Orthodox Christians. By emphasising the primacy of the spirit over the body, Orthodoxy initially disparaged the value of sport and physical culture, but it has evolved to become one of the foremost promoters of physical culture among the faithful in more modern times (Stefanović et al., 2018), as was the case for Greek Christians in the early twentieth century living on the west coast of Asia Minor (Albanidis, 2011).

#### Islam

As in the case of Christianity, the approach of Islam to the issues of the body, corporality and physical activity, broadly understood, is not straightforward. This approach depends on historical, geographical and cultural contexts, a specific theological doctrine (Barlas, 2011) and the interpretation of Sharia pertaining to the body, corporeality and sexuality (Kutscher, 2011) as well as to sport as “inherently socioreligious” (Walseth & Fasting, 2003). Generally, however, man, including his flesh, is the highest act of the Creator. Hence, the Koran and Muslim tradition (hadith) emphasise the importance of health, a healthy lifestyle and care for the body, which was encouraged by Prophet Muhammad himself (al-Khayat, 2004; Tober & Budiani, 2007). While in relation to men, physical activity has never aroused major reservations (on the contrary, the Koran endorsed physical activity in order that men would be ready to fight to defend the faith (Baidruel Hairiel Abd et al., 2019), the participation of women continues to provoke numerous discussions and controversies, such as with respect to the imposition of various prescriptions or proscriptions, for example, the requirement to cover their bodies or inhibitions of their functioning in public spaces (Malchrowicz-Mosko, 2021)). Ostensibly to protect women from temptation and sin, patriarchal Muslim culture forces women to cover their bodies, which makes it difficult to engage in sports. A woman’s body is treated as the property of her family and the community, whose honour is valued more highly than women’s desires and ambitions; hence, a woman in a patriarchal Muslim society must protect her body at all costs (Medina, 2014). Such attitudes can also be encountered among traditionalist Muslim communities in the West, where Muslim women practising sports are viewed as a way of overcoming moral barriers that are imposed by the patriarchal culture. Muslim women who practice sports and come from traditionalist communities and families are often exposed to various types of harassment (Walseth, 2006). Some Muslim intellectuals, especially Muslim feminists, oppose the identification of these restrictions with the Koran, emphasising that such limitations have a cultural (rather than religious) source (Pfister, 2010). There have also been change in perspective among the followers of certain orthodox factions of Islam, such as Saudi Wahhabism. According to the research of Al Ruwaili (2020), which was conducted in the Saudi city of Dammam, the majority of respondents gave an affirmative answer to the question of whether women should be encouraged to practise sports. Most positive answers to this question were provided by the group of men with university educations—70%—and the group of women without university educations—66%. Paradoxically, women with university education were more restrained regarding this matter. However, many Saudis continue to oppose the notion of women playing sports. In their opinion, female sports violate the teachings of Islam and contradict traditional morality.

## Coronavirus Anxiety and Pro-health Behaviours

Unfortunately, pro-health behaviours are often incorporated into individuals’ lifestyles only in the context of health emergencies. Many people begin to follow the principles of a healthy lifestyle only when their health condition is poor or threatened—the fear of losing their health motivates them to begin to care for their health (Trovato, 2012). Unexpectedly, a new fear has emerged and affected nearly the entire world—the coronavirus pandemic (COVID-19). From December 2019 to May 2022, 514 million people fell ill, and over six million deaths have been reported globally (WHO, 2022). The rapidity of COVID-19 has led to threats to mental health, especially with respect to increasing negative emotions and decreasing positive emotions (Nivette et al., 2021; Rania et al., 2022). The prevalence of COVID-19 and the unpredictability of the associated course of infection have resulted in a significant increase in risk perceptions. Risk perception, which refers to one’s beliefs regarding one’s personal susceptibility to a negative event (e.g. Champion & Skinner, 2008), is often considered to be a motivating factor with respect to changes in health behaviours (Gaube et al., 2019).

## Aims and Objectives of the Present Study

**Fig. 1:**
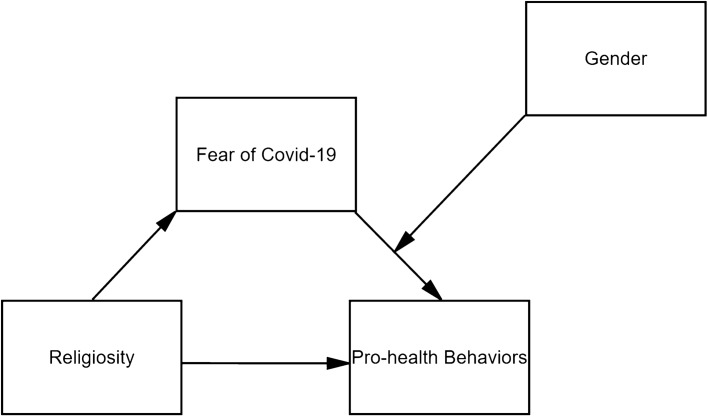
Moderated mediation model tested in the study

The current study specifically constructs a moderated mediation model to define the relationship between religiosity and health-promoting care for the body in the contexts of Christianity and Islam during the COVID-19 pandemic (Fig. 1).

According to the proposed model, the following hypotheses were tested separately for each religion:

### Hypothesis 1

*Coronavirus anxiety mediates the relationship between religiosity and pro-health behaviours (physical activity and diet)*.

### Hypothesis 2

*Gender moderates the indirect relationship between COVID-19 anxiety and pro-health behaviours (physical activity and diet)*.

## Method

### Participants

In total, data from one thousand five hundred and two persons were referenced by the study. To ensure that no intranation interactions would bias the data, we selected three religious groups in accordance with three nationalities. We chose Egypt, Poland, and Romania as countries that exhibit high religiosity indices (Coutinho, 2016) and that feature one dominant religion. Thus, for Sunni Islam, we examined data collected in Egypt, for Roman Catholicism, we examined data from Poland, and for Orthodox Christianity, we examined data from Romania. Detailed information on the demographic characteristics of the participants can be found in Table 1.

**Table 1:** Age of participants

	Gender			Age			
	Women	Men	No answer	Min	Max	*M*	*SD*
Sunni Islam	607	191	–	18	41	19.80	1.46
Roman Catholicism	339	104	–	18	74	33.03	11.95
Orthodox Christianity	201	58	2	18	74	34.63	11.63

### Procedure

The procedure conducted in this study consisted of an online survey that served as part of an international research project registered in the Protocol Registration and Results System—ClinicalTrials.gov https://clinicaltrials.gov/ct2/show/NCT04432038. This study used data collected during the first lockdown resulting from the COVID-19 pandemic among Egyptian, Polish and Romanian populations. The work described was carried out in accordance with the Code of Ethics of the World Medical Association (Declaration of Helsinki) for data collection experiments involving humans. The protocols used in this study were approved by the Ethics Board for Research Projects at the Institute of Psychology, University of Gdansk, Poland (decision no. 33/2020). The questionnaires used for this study took approximately 10 min to complete.

We collected several different kinds of information using the *Coronavirus Anxiety Scale,* the *Eating Attitudes Test,* the *Inventory of Physical Activity Objectives,* two single-item measures of religious denomination and religiosity, and a questionnaire concerning sociodemographic data.

### Instruments

*Religious denomination and religiosity* Religious denomination was measured using one item, “What is your current religion, if any?” To answer this item, respondents chose one of twelve categories. Religiosity was also measured using one item, “How religious do you consider yourself to be?” Response options for this item ranged from 1 = “not at all religious” to 7 = very religious”.

*The Coronavirus Anxiety Scale* (CAS) was developed by Lee (2020) translated into Arabic by (Lee, 2020) and Polish by Skalski et al. (2020). The Romanian version of this scale demonstrated good psychometric properties in a previously published study (Enea et al., 2021). This scale is a five-item self-report questionnaire that allows for the determination of the intensity of dysfunctional anxiety associated with the coronavirus crisis (COVID-19). CAS is a single-factor instrument, in which a higher score indicates a higher level of anxiety on the part of the respondent. The individual items in the questionnaire are rated on a 5-point Likert scale with answers ranging from 0 = “not at all” to 4 = “almost daily”; answers are based on personal experience over the past 2 weeks. The scaling format follows the cross-sectional symptom measure suggested in DSM-5. A total CAS score of ≥ 9 is interpreted as indicating probable coronavirus-related dysfunctional anxiety and warrants further clinical evaluation and/or treatment. In the present study, the Cronbach’s alpha coefficients for the national version of CAS were as follows: 0.89 for Egypt, 0.79 for Poland, and 0.87 for Romania.

*The Eating Attitudes Test* (EAT-26) developed by Garner et al. (1982) is one of the most useful instruments for assessing attitudes and behaviours that are characteristic of eating disorders in both clinical and nonclinical samples and has previously been translated into many languages, including Arabic (al-Subaie et al., 1996; Latzer et al., 2009), Polish (Rogoza et al., 2016) and Romanian (Iconaru & Ciucurel, 2009). This instrument is composed of 26 items to which participants respond on a 6-point Likert-type scale, which is scored as 3 (always), 2 (usually), 1 (sometimes) or 0 (rarely, very rarely or never); the higher an individual’s score is, the more abnormal that individual’s eating habits, which are associated with increased risk of developing eating disorders. To collect data specific to the pandemic period, the questionnaire was preceded by the following instruction: “Please try to answer from the current perspective of the ongoing COVID-19 pandemic”. In our research, this questionnaire was characterized by good reliability indicated by the Cronbach’s αs coefficients: 0.88 in Egyptian, 0.87 in Polish and 0.83 in Romanian samples.

*The Inventory of Physical Activity Objectives* (IPAO) developed by Lipowski and Zaleski (2015) allows for the determination of the individual’s level of involvement in PA. In the standard version of the IPAO, the respondent answers questions regarding their involvement in competitive sports (both previous and current) and the forms and intensity of their physical activity. As a result of the COVID-19 pandemic, questions regarding physical activity were limited to one item, “Do you engage in physical activity during the COVID-19 pandemic?”, which featured two response options: “yes” or “no”.

## Results

*Descriptive statistics* Means, standard deviations and percentages across groups are reported in Table 2.

**Table 2:** Descriptive statistics and zero-order correlation coefficients across studied religious denominations

	*M*	*SD*	1	2	3	4	5
*Sunni*							
1. Age							
2. Gender			–.09^**^				
3. Religiosity	4.89	1.30	–.12^**^	–.00			
4. Fear of Covid-19	1.66	0.85	.04	.12^**^	.00		
5. EAT	4.22	0.75	–.03	–.01	–.03	–.42^**^	
6. Physically active	37.5%		–.06	.22^**^	.09^**^	.18^**^	–.18^**^
*Catholic*							
1. Age							
2. Gender			–.03				
3. Religiosity	4.21	1.63	–.00	.07			
4. Fear of Covid-19	1.59	0.68	–.04	–.18^**^	.12^*^		
5. EAT	4.54	0.59	.19^**^	.30^**^	.02	–.30^**^	
6. Physically active	68.4%		–.15^**^	.01	–.03	–.15^**^	–.09
*Orthodox*							
1. Age							
2. Gender			–.07				
3. Religiosity	4.35	1.62	.16^**^	.08			
4. Fear of Covid-19	1.33	0.59	–.06	–.03	–.07		
5. EAT	4.52	0.57	.04	.17^**^	.09	–.31^**^	
6. Physically active	51.0%		–.22^**^	.04	–.08	–.09	–.15^*^

### Mediation Assumptions Tests

To test the theoretical mediation model described above, we first verified the basic assumptions of the mediational model (MacKinnon et al., 2007) related to (1) the significant effect of independent variable *X* on mediator *M*, namely, the *a* path, and (2) the significant effect of mediator *M* on dependent variable *Y* while controlling for independent variable *X*, namely, the *b* path. The effects required to test the significance of the *a* path are presented in Table 1, which reports zero-order correlation coefficients. Coronavirus anxiety was significantly related to religiosity only among Catholics. Thus, we tested the mediation model only by reference to that group.

### Relationship Between Fear of COVID-19 and Eating Behaviours Across Religions

We began by conducting two hierarchical multiple linear regression analyses—one among Orthodox Christians and one among Sunni Muslims. Eating behaviours were used as the dependent variable in both analyses. As a first step, we entered religiosity and fear of COVID-19 as orthogonal predictors (see Table 2), while gender and age were entered as covariates. In a second step, to examine whether fear of COVID-19 is linked to eating behaviours differentially among men and women, we entered an interaction term related to fear of COVID-19 × gender. Given the fact religiosity and fear of COVID-19 were related among Christians, we tested a moderated mediation model by reference to this group. Although this model was similar to the model estimated for the other two groups, in this context, we additionally tested the indirect effect of religiosity on eating behaviours via fear of COVID-19 using the Process Macro (Model 14, 5000 bootstrap samples; Hayes, 2015). Prior to these analyses, for the purpose of avoiding multicollinearity, we centred all variables (i.e. we subtracted the group-level mean score from the individual score of every participant so that the individual score represented that individual’s deviation from the group average). We found no violations with respect to multicollinearity (max VIF = 1.41), heteroscedasticity, or dependent variable residual normality. We employed the *R*^2^ difference test and the significance of the interaction term as indicators of meaningful differences between genders in terms of the relation between fear of COVID-19 and eating behaviour.

Detailed results of the regression analyses are presented in Table 3. Fear of COVID-19 was related to abnormal eating habits negatively among all religious groups included in the study. Such fear was higher among men but only for the Christian groups (i.e. Orthodox Christians and Catholic). Age was positively related to eating behaviour (i.e. such behaviour increased with respondents’ age) only among the Christian groups. Religiosity was not related to eating behaviour directly for any of the religions, although an indirect effect was found among Catholic women, *β* = − 0.012 [− 0.024, − 0.004] (but not men, *β* = − 0.006 [− 0.023, 0.003]. Nonetheless, these effects were weak and exhibited little variation (index of moderated mediation = 0.006 [− 0.008, 0.020]; Hayes, 2015); accordingly, we treated these effects as psychologically insignificant. Gender did not moderate this effect for either of the two other religious groups. Finally, we compared the strength of the regression coefficients using the *Z* test. The results of this test indicated that the relationship between fear of COVID-19 and eating behaviours was stronger for Sunni Muslims than for Catholic (*Z* = 3.44, *p* < 0.001) or Orthodox Christians (*Z* = 2.10, *p* = 0.018), but no significant differences were found between Catholic and Orthodox Christian respondents (*Z* = − 0.69, *p* = 0.245).

**Table 3:** Religiosity and fear of COVID-19 as predictors of eating behaviours

Step 1	Orthodox Christians			Sunni Muslims			Catholic Christians		
	*R*^2^ = .12 *F*(4, 253) = 8.82 *p* < .001			*R*^2^ = .18 *F*(4, 253) = 43.96 *p* < .001			*R*^2^ = .19 *F*(4, 253) = 25.58 *p* < .001		
	*β*	*t*	*p*	*β*	*t*	*P*	*β*	*t*	*p*
Gender ^a^	**.16**	**2.62**	**.009**	.04	1.30	.195	**.26**	**5.96**	** < .001**
Age	.03	0.44	.657	–.02	–0.55	.580	**.19**	**4.39**	** < .001**
Religiosity	.05	.886	.376	–.03	–1.03	.302	.03	0.64	.524
Fear of C-19	**–.30**	**–5.00**	** < .001**	**–.43**	**–13.17**	** < .001**	**–.25**	**–5.63**	** < .001**

Step 2	Δ*R*^2^ = .01 *F*(4, 253) = 3.04 *p* = .083			Δ*R*^2^ = .00 *F*(4, 253) = 0.42 *p* = .516			Δ*R*^2^ = .00 *F*(4, 253) = 1.20 *p* = .274		
	*β*	*t*	*p*	*β*	*t*	*p*	*β*	*t*	*p*
Gender ^a^	**.15**	**2.57**	**.011**	.05	1.36	.174	**.27**	**6.05**	** < .001**
Age	.02	0.39	.694	–.02	–0.57	.573	**.19**	**4.34**	** < .001**
Religiosity	.04	0.63	.531	–.03	–1.04	.298	.03	0.73	.466
Fear of C-19	**–.25**	**–3.87**	** < .001**	**–.41**	**–10.39**	** < .001**	**–.27**	**–5.54**	** < .001**
Interaction	–.11	–1.74	.083	–.03	–0.65	.516	.06	1.10	.274
Full model	*R*^2^ = .13 *F*(4, 253) = 7.72 *p* < .001			*R*^2^ = .18 *F*(4, 253) = 35.23 *p* < .001			*R*^2^ = .19 *F*(4, 253) = 20.71 *p* < .001		

^a^0 = women, 1 = men. Positive coefficients refer to higher scores among men. C-19 = COVID-19. Significant coefficients are in bold

### Relationship Between Fear of COVID-19 and Physical Activity Across Religions

To test the relationships among religiosity, fear of COVID-19 and physical activity (PA), we conducted multiple hierarchical logistic regression (since physical activity was measured as a binary variable). We also tested for moderated mediation (using Process Macro Model 14 in SPSS software) by reference to Catholics, given the fact that the link between religiosity and fear of COVID-19 was significant only for that group. Detailed results are presented in Table 3. The overall number of people who were physically active was less than 50% among Sunni Muslims, more than 50% among Catholics and approximately 50% among Orthodox Christians (see Table 2). Fear of COVID-19 was a significant predictor of PA among Sunni Muslims and Catholics (but not Orthodox Christians). These effects, however, indicated the opposite direction. Specifically, this effect was positive (i.e. higher fear was linked to a higher probability of PA) among Sunni and negative (i.e. higher fear was linked to a lower probability of PA) among Catholics. Religiosity was positively and directly related to PA among Sunni Muslims (i.e. higher religiosity was linked to a higher probability of PA). Resembling the results concerning eating behaviours, religiosity also exhibited a negative indirect relation to PA among Catholic women, *B* = − 0.02 [− 0.005; − 0.002] (but not men, *B* = − 0.032 [− 0.090; 0.007]); however, these effects were weak and exhibited little variation (index of moderated mediation = − 0.010 [− 0.065; 0.036]. Age was negatively related to PA (i.e. the probability of PA was higher at lower ages) among Orthodox Christians and Catholics. Gender was related to PA only among Sunni Muslims, thus indicating that the probability of PA was higher among men. Gender did not moderate the relationship between fear of COVID-19 and PA for any of the samples (Table 4).

**Table 4:** Religiosity and fear of COVID-19 as predictors of physical activity

Step 1	Orthodox Christians			Sunni Muslims			Catholic Christians		
	*R*^2^ = .09 χ^2^ = 17.23 *p* = .002			*R*^2^ = .11 χ^2^ = 65.72 *p* < .001			*R*^2^ = .06 χ^2^ = 20.38 *p* < .001		
	Odds ratio	*Wald*	*p*	Odds ratio	*Wald*	*p*	Odds ratio	*Wald*	*p*
Gender^a^	1.14	0.17	.677	**2.63**	**30.69**	** < .001**	.901	0.17	.680
Age	**.959**	**12.25**	** < .001**	.948	0.88	.347	**.972**	**11.18**	**.001**
Religiosity	.944	0.51	.477	**1.16**	**6.41**	**.011**	.991	0.02	.890
Fear of C-19	.677	3.02	.082	**1.49**	**19.61**	** < .001**	**.621**	**9.86**	**.002**

Step 2	Δ*R*^2^ = .00 χ^2^ = 0.42 *p* = .516			Δ*R*^2^ = .00 χ^2^ = 0.32 *p* = .572			Δ*R*^2^ = .00 χ^2^ = 0.26 *p* = .610		
	Odds ratio	*Wald*	*p*	Odds ratio	*Wald*	*p*	Odds ratio	*Wald*	*p*
Gender^a^	1.13	0.16	.691	**2.66**	**31.14**	** < .001**	.883	0.234	.629
Age	**.959**	**12.38**	** < .001**	.948	0.89	.344	**.972**	**11.03**	**.001**
Religiosity	.937	0.63	.427	**1.16**	**6.36**	**.012**	.988	0.03	.858
Fear of C-19	.724	1.78	.182	**1.54**	**15.80**	** < .001**	**.645**	**6.70**	**.010**
Interaction	.686	0.42	.519	.90	0.32	.570	.823	0.26	.611
Full model	*R*^2^ = .09 χ^2^ = 17.65 *p* = .003			*R*^2^ = .11 χ^2^ = 66.04 *p* < .001			*R*^2^ = .06 χ^2^ = 20.64 *p* = .001		

^a^0 = women, 1 = men. Positive coefficients refer to higher scores among men. C-19 = COVID-19. *R*^2^ refers to Nagelkerke’s *R*^2^. The constant coefficient in Step 0 refers to the basic probability of performing physical activity in a given group

## Discussion

### Fear of COVID-19 and Eating Behaviours as a Display of Bodily Care Across Religions

To date, some studies have linked pandemic stress and concerns regarding COVID-19 with religiosity and mental health (Counted et al., 2022; Dein et al., 2020; Dobrakowski et al., 2021), especially with respect to religious ways of coping with fear and anxiety, which seem to be related to the topic of mental health in the middle of a pandemic (Counted et al., 2022; Perry et al., 2020).

In a situation featuring fear of a threat that is difficult to control, eating may serve as a compensatory behaviour, i.e. a means of escape from the problem, and so eating may take abnormal forms such as overeating or, in contrast, control of eating, such that paying more attention to proper nutrition may give the appearance that one is able to maintain control over the overall threat (Levinson & Rodebaugh, 2012). Improper eating behaviour can often lead to eating disorders, and this relationship is an example of the fact that caring for or attention to the body can result in mental health problems (Izydorczyk, 2022; Izydorczyk et al., 2021). In our research, the analysis of eating habits (mean values for the overall score on the EAT 26 scale) confirms that among all three groups of respondents (Orthodox Christians, Catholics and Sunni Muslims), no intensification of eating behaviour in a manner typical of the occurrence of eating disorders can be found. The fear of COVID-19, as the fear of a pandemic, has the character of existential anxiety (Schou-Bredal et al., 2021; Scrima et al., 2022), so its influence on the functioning of the individual cannot be compared clearly with the influence of other threatening situations. Analysing the relationship between pandemic anxiety, mood and eating disorders among students, De Pasquale et al. (2021) found that fear of COVID-19 infection is a significant cause of mental disorders during the pandemic. These authors noted that eating is a compensatory experience that can divert attention away from experiences of uncertainty, fear and despair and thus cause changes in eating habits and behaviours. The results concerning the Eating Disorder Inventory (EDI-2) scales and the Binge Eating Scale (BES) indicated that all emotional states measured with the Profile of Mood States (POMS) questionnaire (i.e. tension, depression, anger, fatigue, confusion) were significantly correlated with fear of COVID-19. Women were more likely to fear COVID-19, exhibiting greater difficulties with respect to all the parameters measured. Other authors have also highlighted the adverse effects of the fear of COVID-19 on eating habits (Amatori et al., 2020; Fernández-Aranda et al., 2020; Huber et al., 2021; Rodgers et al., 2020; Touyz et al., 2020), emphasising the need to control for additional variables such as health-promoting behaviours, somatic comorbidities and mental health. Our research, however, showed that greater fear of infection was associated with lower tendencies to follow a strict diet or overeat among respondents of all religions. Nevertheless, the strongest and most negative relationship between the fear of COVID-19 and improper eating behaviour was noted for the Sunni group. No previous studies have been conducted to measure the relationship between COVID-19 anxiety and eating behaviour simultaneously while taking into account people’s specific religious beliefs, as accomplished by the authors of this article. Therefore, it is difficult to compare the results of this study with the results found by other authors in this field of research. Perry et al. (2020), who analysed data collected during the second wave of the pandemic, found that Christian nationalism was a leading predictor of Americans’ engagement in careless behaviours, such as eating in restaurants, and religiosity was the leading predictor of Americans’ likelihood of engaging in preventative behaviours. These results seem to confirm our assumption that not only people’s type of religion but also their levels of religiosity can be important in this context. Contrary to the assumptions adopted for our article, people’s level of religiosity was not a significant mediator of the relationship between fear of COVID-19 and compensatory eating. Additionally, the gender of the respondents was not significant, i.e. no differences in the strength of the indirect relationships between women and men could be found.

### The Fear of COVID-19 and Involvement in Pro-health Physical Activity Across Religions

The results of the current study showed that Catholics were the most physically active group rather than Sunni Muslims or Orthodox Christians and that fear of COVID-19 was a significant predictor of PA among Sunni and Catholics but not among Orthodox Christians. Interestingly, higher fear was linked to a higher probability of PA among Sunni, but this relationship took the opposite direction among Catholics, among whom higher fear was linked to a lower probability of PA. According to the studies by Merrill and Thygerson (2001) among religious groups (Catholics and Mormons), those who attend church weekly are more likely to exercise than individuals who attend church less often than weekly. In studies of adult Arab migrant populations, El Masri et al. (2021) highlighted the fact that other unique factors that are closely associated with culture and religion appear to influence the levels of physical activity among such migrants compared to other religious populations. However, as the current study shows, the probability of exercising is also linked to COVID-19 anxiety. In studies of Polish and Chinese respondents, Wilczyńska et al. (2021) proved that fear of COVID-19 changes people’s motives to engage in PA and that these motives vary between women and men as well as among people of different ages. Additionally, the results of this study indicated that age was negatively related to PA among Orthodox Christians and Catholics and that gender was related to PA only among Sunni Muslims, also indicating that the probability of PA was higher among men, which is in contradiction with the results of many other studies showing a different approach to health-promoting physical activity based on gender (Pawłowska et al., 2021). Kahan (2018), investigating advertisements of PA programming in Islamic centres in the USA, noted that most activities—with the exception of fitness classes—were advertised to a male audience and underlined the fact that there is a gap with respect to advertisements targeting women, which could be a reason underlying the fact that fewer women who following this particular religion engage in exercise. These differences are culturally determined. The female body in the context of PA could be treated as *Haram* by Muslim traditions. Of course, not all Muslims regard sports practised by women as *Haram ex definitione,* but some elements of PA are strictly restricted by Islamic rules regarding women’s clothes or behaviours. These restrictions effectively limit the ability of women to engage in PA (Malchrowicz-Mosko, 2021).

## Conclusions and Limitations

Fear of COVID-19 is associated with engagement in pro-health activity, although not to such a significant extent as might be expected. The type of religion in question was revealed to moderate this relationship, but the intensity of religiosity was not found to serve as a moderator. A limitation as well as an advantage of this research is its short timeframe for collecting data—the period of the so-called first wave of COVID and the related universal and strictly observed lockdown. Especially with regard to PA, COVID-19 limitations are an essential factor. The task of extending this analysis by reference to data taken from subsequent “covid waves” would be worthwhile.

## References

[CR1] Baidruel Hairiel Abd R, Nurazzura Mohamad D, Haizuran Mohd J, Abdul Sham A (2019). Islam and sport: From human experiences to revelation. Intellectual Discourse.

[CR2] Abdala GA, Meira MDD, Rodrigo GT, Fróes MBDC, Ferreira MS, Abdala SA, Koenig HG (2021). Religion, age, education, lifestyle, and health: structural equation modeling. Journal of Religion and Health.

[CR3] Albanidis E (2011). Athletics activities of the greek community in smyrna from the end of the 19th century to 1922. Studies in Physical Culture & Tourism.

[CR4] Al-Khayat MH (2004). Health as a human right in Islam.

[CR5] Al-Ruwaili, M. D. (2020). *Females and sport in Saudi Arabia: An analysis of the relationship between sport, region, education, gender, and religion. PhD Thesis.* University of Stirling. http://hdl.handle.net/1893/31239

[CR6] Al-Subaie A, Al-Shammari S, Bamgboye E, Al-Sabhan K, Al-Shehri S, Bannah AR (1996). Validity of the arabic version of the eating attitude test. International Journal of Eating Disorders.

[CR7] Amatori S, Donati Zeppa S, Preti A, Gervasi M, Gobbi E, Ferrini F, Rocchi MBL, Baldari C, Perroni F, Piccoli G, Stocchi V, Sestili P, Sisti D (2020). Dietary habits and psychological states during COVID-19 home isolation in italian college students: The role of physical exercise. Nutrients.

[CR8] Barlas A (2011). Abraham's sacrifice in the Qur’an: Beyond the body. Scripta Instituti Donneriani Aboensis.

[CR9] Champion VL, Skinner CS, Glanz K, Rimer BK, Viswanath K (2008). The health belief model. Health behaviour and health education. Theory, research, and practice.

[CR10] Counted V, Pargament KI, Bechara AO, Joynt S, Cowden RG (2022). Hope and well-being in vulnerable contexts during the COVID-19 pandemic: Does religious coping matter?. Journal of Positive Psychology.

[CR11] Coutinho JP (2016). Religiosity in Europe: An index, factors, and clusters of religiosity. Sociologia, Problemas e Práticas.

[CR12] Curran CE (2019). Diverse voices in modern US moral theology.

[CR13] De Pasquale C, Sciacca F, Conti D, Pistorio ML, Hichy Z, Cardullo RL, Di Nuovo S (2021). Relations between mood states and eating behavior during COVID-19 pandemic in a sample of italian college students. Frontiers in Psychology.

[CR14] Dein S, Loewenthal K, Lewis CA, Pargament KI (2020). COVID-19, mental health and religion: An agenda for future research. Mental Health, Religion & Culture.

[CR15] Dobrakowski PP, Skalski S, Surzykiewicz J, Muszyńska J, Konaszewski K (2021). Religious coping and life satisfaction during the COVID-19 pandemic among polish catholics. The mediating effect of coronavirus anxiety. Journal of Clinical Medicine.

[CR16] El Masri A, Kolt GS, George ES (2021). A systematic review of qualitative studies exploring the factors influencing the physical activity levels of Arab migrants. International Journal of Behavioral Nutrition and Physical Activity.

[CR17] Enea V, Candel OS, Zancu SA, Scrumeda A, Bărbuşelu M, Largu AM, Manciuc C (2021). Death anxiety and burnout in intensive care unit specialists facing the COVID-19 outbreak: The mediating role of obsession with COVID-19 and coronaphobia. Death Studies.

[CR18] Fernández-Aranda F, Casas M, Claes L, Bryan DC, Favaro A, Granero R, Gudiol C, Jiménez-Murcia S, Karwautz A, Le Grange D, Menchón JM, Tchanturia K, Treasure J (2020). COVID-19 and implications for eating disorders. European Eating Disorders Review.

[CR19] Garner DM, Olmsted MP, Bohr Y, Garfinkel PE (1982). The eating attitudes test: Psychometric features and clinical correlates. Psychological Medicine.

[CR20] Gaube S, Lermer E, Fischer P, Raue M, Streicher B, Lermer E (2019). The concept of risk perception in health-related behavior theory and behavior change. Perceived safety: A multidisciplinary perspective.

[CR21] Hayes AF (2015). An index and test of linear moderated mediation. Multivariate Behavioral Research.

[CR22] Heszen-Celińska I, Sęk H, Heszen-Celińska I, Sęk H (2020). Koncepcje i pojęcie zdrowia w psychologii i naukach o człowieku. PsychologiazZdrowia [Health Psychology].

[CR23] Huber BC, Steffen J, Schlichtiger J, Brunner S (2021). Altered nutrition behavior during COVID-19 pandemic lockdown in young adults. European Journal of Nutrition.

[CR24] Iconaru EI, Ciucurel C (2009). The relation between the dietary habits and choices, the nutritional status and the physical activity regime in Romanian adolescents. Journal of Physical Education and Sport.

[CR25] Izydorczyk B (2022). Body image in eating disorders: Clinical diagnosis and integrative approach to psychological treatment.

[CR26] Izydorczyk B, Truong Thi Khanh H, Lipowska M, Sitnik-Warchulska K, Lizińczyk S (2021). Psychological risk factors for the development of restrictive and bulimic eating behaviors: A polish and vietnamese comparison. Nutrients.

[CR27] Jacobson HL, Hall MEL, Anderson TL, Willingham MM (2016). Temple or prison: Religious beliefs and attitudes toward the body. Journal of Religion and Health.

[CR28] Jonveaux I, Giordan G, Pace E (2012). Mapping religion and spirituality in a postsecular world. Asceticism and the place of the body in modern monastic prayer.

[CR29] Kahan D (2018). Physical activity programming advertised on websites of U.S. Islamic centers: A content analysis. International Journal of Environmental Research and Public Health.

[CR30] Kutscher J (2011). Towards a solution concerning female genital mutilation? An approach from within according to Islamic legal opinions. Scripta Instituti Donneriani Aboensis.

[CR31] Latzer Y, Azaiza F, Tzischinsky O (2009). Eating attitudes and dieting behavior among religious subgroups of Israeli-Arab adolescent females. Journal of Religion and Health.

[CR32] Lee SA (2020). Coronavirus anxiety scale: A brief mental health screener for COVID-19 related anxiety. Death Studies.

[CR33] Levinson CA, Rodebaugh TL (2012). Social anxiety and eating disorder comorbidity: The role of negative social evaluation fears. Eating Behaviors.

[CR34] Lipowski M, Zaleski Z (2015). Inventory of physical activity objectives – a new method of measuring motives for physical activity and sport. Health Psychology Report.

[CR35] MacKinnon DP, Fairchild AJ, Fritz MS (2007). Mediation analysis. Annual Review of Psychology.

[CR36] Malchrowicz-Mosko E (2021). Can she run or scream while travelling? Is sports tourism halal or haram? Management of sports travel of muslim women in the era of west-east dichotomy. European Research Studies Journal.

[CR37] McKay R, Whitehouse H (2015). Religion and morality. Psychological Bulletin.

[CR38] Medina J (2014). This battlefield called my body: Warring over the Muslim female. Religions.

[CR39] Merrill RM, Thygerson AL (2001). Religious preference, church activity, and physical exercise. Preventive Medicine.

[CR40] Nivette A, Ribeaud D, Murray A, Steinhoff A, Bechtiger L, Hepp U, Shanahan L, Eisner M (2021). Non-compliance with COVID-19-related public health measures among young adults in Switzerland: Insights from a longitudinal cohort study. Social Science & Medicine.

[CR41] Norenzayan A (2014). Does religion make people moral?. Behaviour.

[CR42] Owen L, Corfe B (2017). The role of diet and nutrition on mental health and wellbeing. Proceedings of the Nutrition Society.

[CR43] Pawłowska A, Lipowska K, Krokosz D (2021). Too masculine for healthcare, too feminine for intense sports: Correlation between gender conformity and pro-health behaviours. Baltic Journal of Health and Physical Activity.

[CR44] Perry SL, Whitehead AL, Grubbs JB (2020). Culture wars and COVID-19 conduct: Christian nationalism, religiosity, and Americans’ behavior during the coronavirus pandemic. Journal for the Scientific Study of Religion.

[CR45] Pfister G (2010). Women and sport in Islamic countries. Forum for Idræt.

[CR46] Rania N, Pinna L, Coppola I (2022). Living with COVID-19: Emotions and health during the pandemic. Health Psychology Report.

[CR47] Rodgers RF, Lombardo C, Cerolini S, Franko DL, Omori M, Fuller-Tyszkiewicz M, Linardon J, Courtet P, Guillaume S (2020). The impact of the COVID-19 pandemic on eating disorder risk and symptoms. International Journal of Eating Disorders.

[CR48] Rogoza R, Brytek-Matera A, Garner DM (2016). Analysis of the EAT-26 in a non-clinical sample. Archives of Psychiatry and Psychotherapy.

[CR49] Schou-Bredal I, Grimholt T, Bonsaksen T, Skogstad L, Heir T, Ekeberg Ø (2021). Optimists’ and pessimists’ self-reported mental and global health during the COVID-19 pandemic in Norway. Health Psychology Report.

[CR50] Scrima F, Miceli S, Caci B, Cardaci M (2022). The relationship between fear of COVID-19 and intention to get vaccinated. The serial mediation roles of existential anxiety and conspiracy beliefs. Personality and Individual Differences.

[CR51] Shilling C, Mellor PA (2014). Re-conceptualizing sport as a sacred phenomenon. Sociology of Sport Journal.

[CR52] Skalski, S., Uram, P., & Dobrakowski, P. (2020). *Coronavirus anxiety scale - polish version*. https://sites.google.com/cnu.edu/coronavirusanxietyproject/home 10.1016/j.paid.2020.110540 PMC7670928 33223590

[CR100] Stefanović Đ, Šiljak V, Mijatović S, Vukašinović V (2018). The orthodoxy and sports. Physical Education and Through the Centuries.

[CR53] Tober DM, Budiani D (2007). Introduction: Why Islam, Health and the Body?. Body & Society.

[CR54] Touyz S, Lacey H, Hay P (2020). Eating disorders in the time of COVID-19. Journal of Eating Disorders.

[CR55] Trovato GM (2012). Behavior, nutrition and lifestyle in a comprehensive health and disease paradigm: Skills and knowledge for a predictive, preventive and personalized medicine. EPMA Journal.

[CR56] van den Heever G (2014). Introduction: Intersections of discourses – pliable body, the making of religion, and social definition. Religion and Theology.

[CR57] Walseth K (2006). Young Muslim women and sport: The impact of identity work. Leisure Studies.

[CR58] Walseth K, Fasting K (2003). Islam’s view on physical activity and sport: Egyptian women interpreting Islam. International Review for the Sociology of Sport.

[CR59] Watson NJ, Parker A, Watson NJ, Parker A (2013). Sport and Christianity: Mapping the field. Sport and Christianity. Historical and contemporary perspectives.

[CR60] Weaver DF (2020). Christian formation and moral pluralism: Challenges and opportunities. Studies in Christian Ethics.

[CR61] WHO. (2020). *World Health Organization guidelines on physical activity and sedentary behaviour*. https://www.who.int/publications/i/item/9789240015128 33369898

[CR62] WHO. (2022). *Weekly epidemiological update on COVID-19 - 11 May 2022*. https://www.who.int/emergencies/diseases/novel-coronavirus-2019/situation-reports

[CR63] Wilczyńska D, Li J, Yang Y, Fan H, Liu T, Lipowski M (2021). Fear of COVID-19 changes the motivation for physical activity participation: Polish-Chinese comparisons. Health Psychology Report.

[CR64] Williamson B (2004). Christian art: A very short introduction.

